# A generic framework for individual-based modelling and physical-biological interaction

**DOI:** 10.1371/journal.pone.0189956

**Published:** 2018-01-19

**Authors:** Asbjørn Christensen, Patrizio Mariani, Mark R. Payne

**Affiliations:** DTU Aqua, Technical University of Denmark, Kgs. Lyngby, Denmark; Universidade de Aveiro, PORTUGAL

## Abstract

The increased availability of high-resolution ocean data globally has enabled more detailed analyses of physical-biological interactions and their consequences to the ecosystem. We present IBMlib, which is a versatile, portable and computationally effective framework for conducting Lagrangian simulations in the marine environment. The purpose of the framework is to handle complex individual-level biological models of organisms, combined with realistic 3D oceanographic model of physics and biogeochemistry describing the environment of the organisms without assumptions about spatial or temporal scales. The open-source framework features a minimal robust interface to facilitate the coupling between individual-level biological models and oceanographic models, and we provide application examples including forward/backward simulations, habitat connectivity calculations, assessing ocean conditions, comparison of physical circulation models, model ensemble runs and recently posterior Eulerian simulations using the IBMlib framework. We present the code design ideas behind the longevity of the code, our implementation experiences, as well as code performance benchmarking. The framework may contribute substantially to progresses in representing, understanding, predicting and eventually managing marine ecosystems.

## Introduction

Over the last years, the research areas addressing physical-biological interaction in the ocean have clearly enjoyed access to an increasing number of data sets (see e.g. [1, 2]) with oceanographic parameters from coupled ocean circulation models with high temporal and spatial resolution. Lagrangian models coupled to ocean circulation data are a popular approach to understand mechanisms of physical-biological interaction, because most biological knowledge is at the individual level, which is very easy to translate into a Lagrangian model, since a Lagrangian model traces the life of a set of individuals. However, the variety of coupled ocean circulation models produces output with very different data layouts. Additionally a highly variable research funding landscape makes long-term marriages between a biological model and a single physical model unsustainable. Thus it has become a challenge to develop flexible, coupled biological models across several projects. One strategy to address this is to have same biological model reimplemented several times in the context of many physical data sets; however it quickly becomes a full time job to add new features to such models, because it needs to be duplicated in many contexts and it becomes difficult to ensure that parallel implementations are numerically equivalent.

A more robust strategy is to abstract output from coupled ocean circulation models or, more plainly spoken, encapsulate details of the model. The advantages of generic interfaces in model coupling has also long been recognized and demonstrated by the pioneering model systems General Estuarine Transport Model (GETM) [3] and Framework for Aquatic Biogeochemical Models (FABM) [4] for coupling lower trophic levels with ocean circulation. In computer science it is well recognized that a key to software longevity is careful design of code layout, following paradigms of object-oriented coding and generic software design patterns [5, 6] In down-stream disciplines, being more focused on results than coding, modern coding principles are less celebrated, partially due to ubiquitous legacy codes. With the ability to seamlessly exchange either physics or biological components in a coupled model system, direct comparisons of models will be feasible without hidden biases. However, such an exercise is quite non-trivial today.

Lagrangian modelling is practiced in very different disciplines in science and Lagrangian modelling in ecology has been reviewed some time ago [7–9]. Lagrangian modelling is also referred to as individual-based, agent-based or ant modelling in ecology. Some efforts have been made to propose minimal standards [10, 11] for reporting Lagrangian modelling, as well as best practice guides [12] for marine ecology. Numerous frameworks for Lagrangian modelling exist, some being developed enthusiastically, others living a quiet life. Here we will only consider Lagrangian modelling frameworks subject to open access and relevant for studying physical-biological interaction in marine ecology. NetLogo [13, 14] is a highly versatile, java-based modelling framework applied to very diverse cases in biology, physics, socioeconomics and basic system dynamics, coming along with a graphical user-interface (GUI) and an extensive model library. Even though NetLogo’s ability to explore emergent propertiers of individual-level mechanisms is obvious, it remains to be demonstrated as an efficient tool for studying physical-biological interaction of millions of particles in relation to time-dependent realistic ocean circulation with a realistic topography. The ARIANE code [15] has a long track record in this respect; ARIANE is a computational tool (Fortran 90/95) that is dedicated to the off-line calculation of 3D streamlines off-line diagnostic tool. *Off-line* mode means that the Lagrangian calculation is based on a precalculated database of ocean current fields; *on-line* mode means that the Lagrangian calculation is run in parallel with the ocean circulation model so that ocean current fields does not have to be stored. ARIANE cannot be used for on-line trajectories computations within an ocean circulation model and only passive biology without internal state variables (or very simple types of active [16] behavior) are supported. ARIANE has been adapted to analyze ROMS outputs, however, according to the user manual most data on an Arakawa C-grid [17] can be used as an input. The Larval TRANSport Lagrangian model (LTRANS) code [18] is an example of a code for Lagrangian modelling originally developed for a specific purpose (oyster larvae in Chesapeake Bay), but later generalized to other contexts, e.g. [19]. It is also an off-line particle-tracking setup in Fortran 90/95 code, which is the still preferred coding language in disciplines neighboring physical oceanography, when high computational performance is needed.

In this work we address the challenges involved in generalizing a specific Lagrangian model to a generalized Lagrangian modelling framework, with emphasis on applications involving physical-biological interaction of large particle ensembles with time-dependent realistic ocean circulation data. The objective of our framework presented below is to fulfill these needs in relation to Lagrangian modelling and studies of physical-biological interaction, based on strict separation(encapsulation) of simulation, biology and oceanography via canonical interfaces and present examples of research applications in ecology. Further biological/physical ensemble runs can be performed quite easily by a single scientist without involving several model operators. This increased flexibility to pair physical and biological models also becomes an important asset in relation to new collaborative projects, making it easier to combine available resources by removing barriers of model coupling. But the overarching challenge here is to ensure data and code abstraction does not impair computational performance.

## Framework description

**Fig 1:**
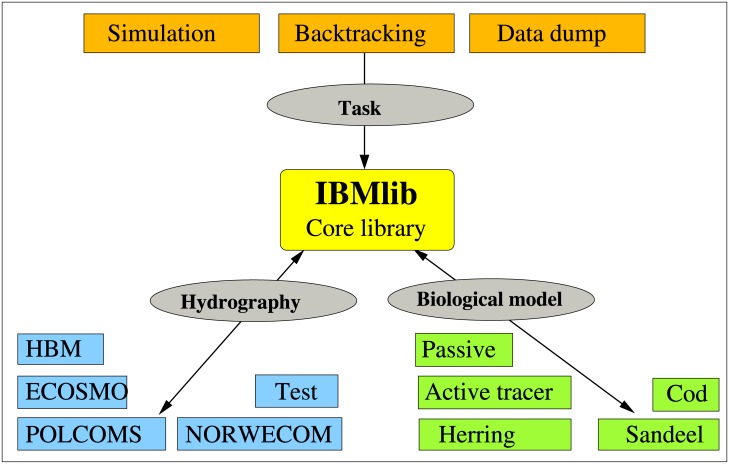
The lego block coupling concept of IBMlib. In a configuration, a physics module (light blue) is combined with a biology module (green) describing an individual organism together with a task (red) controlling the overall computational flow. The light blue physics modules refer to examples of physical/biogeochemical model with an interface to IBMlib: the HIROMB Baltic Model (HBM) [20–22], the ECOSystem MOdel (ECOSMO) [23, 24], the NORWegian ECOlogical Model (NORWECOM) [25, 26] and the Proudman Oceanographic Laboratory Coastal Ocean Modelling System (POLCOMS) [27, 28]. Modules “Test” refer to idealized hydrographical flows and data used for developing/validating/analyzing biological models.

In this work we introduce the Individual-Based Modelling library (IBMlib) software package for agent-based simulations and physical-biological interaction assessment. Details about software availability and requirements are provided in S1 Appendix. The overall purpose of IBMlib is to offer a highly versatile, well-tested platform for Lagrangian simulations and other methodologies addressing physical-biological interaction to academic research and scientific decision support analyzes. The overall concept and layout of IBMlib is shown in Fig 1. The main idea is that a biology and a physics module (selected from a provided data base, possibly customized as needed), are combined with a task module that organizes the overall computational flow, corresponding to the desired output. Examples of tasks are Lagrangian simulations, data extractions or connectivity calculations. Thereby a huge array of problems can be addressed. The task module is combined with (i) a biology module defining properties of Lagrangian particles in the simulation, e.g. behavior (e.g swimming), internal states (e.g size and weight) and the dynamics of internal states, and (ii) a physics module providing access to the local physical/biogeochemical environment of Lagrangian particles.

This layout has proved very robust for 5+ years in very different project contexts. To facilitate their role, modules (i) and (ii) provides standardized interfaces, the biological and physical interface, respectively. The standardization means that either the biological and/or physical module can seamlessly be replaced with alternatives, a core requirement for conducting model ensemble runs. The standardized biological and physical interfaces are merged with other services provided by the IBMlib core at build time, all together forming the IBMlib application programming interface (API). At a coding level, implementation of an interface means writing some subroutines and data type definitions according to a template.

### Task modules

Task modules manages the overall flow of the desired type of simulation. The following task modules have been implemented:

- Lagrangian simulations / backtracking. The core task of IBMlib is to support Lagrangian simulations for an ensemble of particles *i* = 1…*N* each with position and state vectors (*x*_*i*_, *s*_*i*_) updated as
  $$\begin{array}{ccc} dx_{i} & = & \left(u_{i}+w_{i}\right)dt+\nabla K_{i}dt+\rho \sqrt{2K(x_{i}+\frac{1}{2}\nabla K_{i}dt)dt} \end{array}$$ (1)
  $$\begin{array}{ccc} ds_{i} & = & G_{i}dt \end{array}$$ (2)
  where *dt* is a small time increment, (*u*_*i*_, *K*_*i*_) are currents and eddy diffusivity (if available) computed by the physics module, and *w*_*i*_ is the active motion component (e.g. swimming or buoyancy), internal state increment rate *G*_*i*_, both computed by the biology module. *ρ* is a normalized random deviate; the latter term in Eq (1) include the diffusivity gradient correction term [29] avoiding artificial particle aggregation. For backtracking there are fundamental limitations on running time backwards and there are different methodologies [30] reflecting different underlying assumptions. IBMlib features the simple adjoint Lagrangian dynamics discussed in Christensen et al. [31], which is founded on physical arguments, where the backward time update is obtained by reversing (*u*_*i*_, *G*_*i*_) which has the advantage of including the effect of small scale eddies (turbulence) on particle backward transport. IBMlib has optional algorithms for higher order integration of particle trajectories to increase time steps. A low-end laptop can easily perform a simulation with ∼ 10^6^ particles with IBMlib, as we will show later.
- Averaging oceanographic parameters over regions and periods. This is done with a task module that samples the physical environment via the physics module as needed. More advanced examples of this is providing physical/biogeochemical input in a special format to other biological models (e.g Atlantis [32])
- Habitat connectivity calculation. A recurring topic in the usage of IBMlib is habitat connectivity calculation from first principles. Lagrangian simulations are very well suited for studying this, because one releases individuals in habitats and sees how they redistribute between habitats and thereby infer connectivity from exchanges. For sedentary species, connectivity is coincident with recruitment probability *T*_*ji*_ from spawning habitat *i* to settlement in habitat *j* assessed by the estimator
  $$\begin{array}{c} T_{ji}=\frac{1}{R_{i}}N_{ji} \end{array}$$ (3)
  where *R*_*i*_ is the number of released propagules from spawning habitat *i* and *N*_*ji*_ is the number of propagules settling habitat *j* originating from *i* (weighted by their survival change along the transport). IBMlib has a task module that automatically computes *T*_*ji*_ from a Lagrangian simulation by Eq (3).
- Eulerian simulations. Another recent experimental extension of the IBMlib framework is posterior Eulerian dynamics where one solves advection-diffusion-reaction type field equations for a property *C* like
  $$\begin{array}{c} \frac{\partial C}{\partial t}=\nabla \cdot \left(uC-K\nabla C\right)+\hat{G}C \end{array}$$ (4)
  in a box geometry, where $\hat{G}$ is a local reaction operator on the property *C*, e.g. describing growth/consumption, and *u* is the current vector field and *K* a local diffusivity.
- Online cast of IBMlib. Allows to plug-in IBMlib into a physical circulation model, and perform e.g. Lagrangian simulations along with the flow field calculation. This experimental usage of IBMlib is elaborated in S3 Appendix.

These tasks represent most common usages, but usually each study has its own twist; rather than shipping along very byzantine task modules, attempting to capture every variant of what users might want to do, the style of IBMlib is to tailor a similar task module to exactly what you want, writing output exactly as you want by adding/modifying/deleting a few lines of Fortran in suitable template task.

### Biology interface

The biology interface gives a portal for managing particle state properties beyond that of a passive point particle; the properties and dynamics of a passive point particle are built into the core IBMlib services as the background (null) model. The biology interface is described in Table 1. This set of subroutine/types allows the IBMlib to decorate the background model (a passive point particle floating with the water) with arbitrary behavior (e.g swimming or homing), and arbitrary internal states (e.g. age, size, condition and ontogenetical stage) as well as time integration of the internal states. This abstraction level accommodates all reasonable biological organisms acting on their own in response to ambient conditions. If IBMlib is used for a data extraction type job, the biology interface is not utilized.

**Table 1:** Upper level biology interface in IBMlib. The interface defines particle behavior, states and dynamics. The derived type state_attributes contains all the internal state variables pertaining to the complexity level organisms are described at.

service	type	summary
state_attributes	derived type definition	definition of internal particle states
init_particle_state	subroutine	Module start-up method
close_particle_state	subroutine	Module close-down method
init_state_attributes	subroutine	Initialize an instances of particle_state
get_active_velocity	subroutine	Behavior velocity component of the particle
update_particle_state	subroutine	Update particle_state instance state
delete_state_attributes	subroutine	state_attributes destructor
write_state_attributes	subroutine	print particle_state content for debugging (optional)

The biological interface interacts with the IBMlib core in a standard Lagrangian simulation; the interface may internally delegate requests to subinterfaces, which are either standardized or customized; current one subinterface exist for describing embedded biological stages, e.g. a larval or an egg stage, as well as settlement dynamics. This is of interest for advanced developments and is elaborated in S4 Appendix.

### Physics interface

The physics interface provides access to the local physical/biogeochemical environment and offers generic query functions for the aquatic environment; the biology module in an IBMlib combination may apply the services offered by the physics interface. Within IBMlib, space is referenced plainly as continuous geographic coordinates (longitude East, latitude North) horizontally and depth below sea surface vertically (the API and code templates supports dynamic sea surface elevation). Time is represented by a derived clock type based on an underlying open source time library [33] offering a variety of calender and time arithmetics. A master clock is held in the physics module for synchronizing physical fields to a lookup-time in a particle simulation or data extraction context. Importantly, query functions hide details about data representation (grid type, resolution, nested grids, vertical layer structure, layout etc) so that biological models do not become entangled with the data representation in particular data sets.

Table 2 defines the basic physical interface and biogeochemical extension. The physics interface works both in off-line and on-line situations; until now however, no realistic case studies have been performed using on-line mode.

**Table 2:** Upper level physics interface. The basic physical interface and current interface extensions in IBMlib. Interface extensions are for specialized cases and need not be implemented for standardized tasks.

service	type	summary
**Basic interface**		
init_physical_fields	subroutine	initialize physics module
close_physical_fields	subroutine	close physics module
update_physical_fields	subroutine	update physics to current time
get_master_clock	function	access current clock
set_master_clock	subroutine	set current clock
interpolate_turbulence	subroutine	access particle diffusivity
interpolate_turbulence_deriv	subroutine	access particle diffusivity derivative
interpolate_currents	subroutine	access local currents
interpolate_wdepth	subroutine	access local water depth
is_wet	function	3D dry/wet query
is_land	function	horizontal land water query
horizontal_range_check	function	horizontal range check
coast_line_intersection	subroutine	detect coastal crossings
**Physics extensions**		
interpolate_temp	subroutine	access local temperature
interpolate_dslm	subroutine	access local sea level elevation
interpolate_windstress	subroutine	access local surface wind stress
interpolate_salty	subroutine	access local salinity
**Biogeochemistry extensions**		
interpolate_zooplankton	subroutine	access local zooplankton concentration
interpolate_oxygen	subroutine	access local O_2_ concentration
interpolate_nh4	subroutine	access local NH_4_ concentration
interpolate_no3	subroutine	access local ${\text{NO}}_{3}^{-}$ concentration
interpolate_po4	subroutine	access local ${\text{PO}}_{4}^{3-}$ concentration
interpolate_diatoms	subroutine	access local diatom concentration
interpolate_flagellates	subroutine	access local flagellate concentration
interpolate_cyanobacteria	subroutine	access local cyanobacteria concentration
interpolate_organic_detritus	subroutine	access local organic detritus concentration
interpolate_part_org_matter	subroutine	access local particular organic matter conc.
interpolate_DIC	subroutine	access local dissolved inorganic carbon conc.
interpolate_alkalinity	subroutine	access local alkalinity
interpolate_DIN	subroutine	access local dissolved inorganic nitrogen conc.
interpolate_chlorophyl	subroutine	access local chlorophyl concentration
**On-line coupling extension**		
link_to_host_data	subroutine	dynamic access to host model

The physics interface is divided into a basic part, needed to perform a standard Lagrangian simulation, and extension parts addressing e.g. biogeochemistry. This distinction is made, first because not all relevant physical data sets provide data to support extension parts, and secondly also to make it easier to add an interface to a new physics data set, if one does not need e.g. biogeochemistry in the Lagrangian dynamics. Of course this means very basic physical data sets can not be combined with some biological modules requiring extension parts of the physical interface. Such a mismatch will be detected at build time, for impossible combinations of physics and biology.

### IBMlib core library design and building

The IBMlib framework is built up as a template design pattern [5]. The combination of physics, biology and task is done at build time for computational efficiency, by selection in a single file. The framework has been compiled to various Linux platforms with different compilers, and cross-compiled to Windows, using the Minimalist GNU for Windows (MinGW) as an experimental usage. A key to this concept is software separability with clear interfaces (APIs) between modules. The IBMlib core library provides generic functionality and delegates sub tasks to the physics/biology modules via the canonical interfaces in Tables 2 and 1:

- Particle ensembles logistics: setup/release
- Particle ensembles dynamics:
  - Combined advection and random-walk
  - Particle propagation using standard time integration schemes
  - Forward/reversed time dynamics
- Miscellaneous utilities like time arithmetics, geometric analysis, basic statistics, constants, I/O

**Fig 2:**
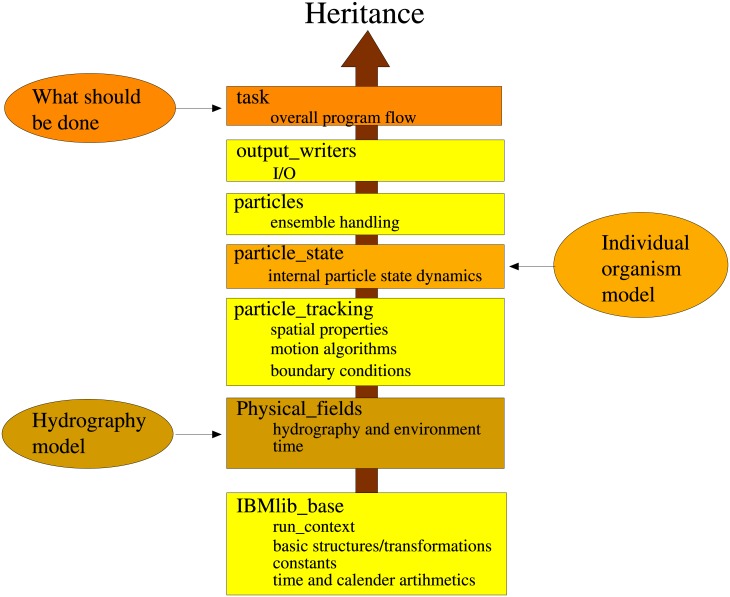
Module dependence hierarchy in IBMlib core. Dependencies and the relation to provider modules (task, biology and physics). An IBMlib configuration is built from the bottom up. Provider modules for physics, biology and task are linked to the IBMlib core (yellow) at build time, forming an executable program.

**Fig 3:**
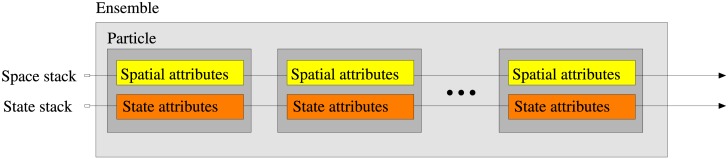
IBMlib particle composition. A particle in IBMlib is a plain composition of a generic spatial_attributes instance and a state_attributes instance, defined by the used biological module.

Standard time integration schemes means Euler-forward and Runge-Kutta variants [34]. The core functionality is organized into logical modules; the module structure is displayed in Fig 2. The modular organization and inheritance structure is shaped by the syntactical requirements of Fortran90. The lowest box in the Fig 2 represents several generic logical modules, which in the figure are coalesced for simplicity. The core class in a Lagrangian setup is the particle class sketched in Fig 3, which in IBMlib is a plain composition of a generic spatial_attributes representing spatial state and location, mobility boundary behavior settings and a user defined state_attributes, which will be empty for passive particles and very advanced for complex organismic models. The set of active particles in a simulation is referred to as a particle ensemble (not to be confused with a model ensemble); usually only one particle ensemble is defined in a simulation, and the number of active particles is defined at run-time by input parameters. All instances of spatial_attributes and state_attributes in the particle ensembles are organized in stacks for computational efficiency, as indicated in Fig 3.

The coding language of the core IBMlib was chosen to be Fortran90+. This language offers of computational speed, simplicity, transparency, compactness, ability to integrate/couple with legacy codes, and fair support for modern principles of object oriented programming and code design [5], including object classes and methods, data encapsulation, polymorphism, and (limited) inheritance. If one wishes to build IBMlib in a combination using already implemented modules for task, physics and biology, all one needs to do is to point to these modules in a configuration file and build the code.

### Data requirements for an IBMlib study

What is needed to get started with an IBMlib study, once the source code has been downloaded from the download site (see S1 Appendix)? This depends on what type of study one is interested in. If the objective is a data extraction, one needs to establish a 3D hydrographic data set with sufficient spatial and temporal resolution and coverage (off-line mode). These data sets may require significant storage space (1-10 TB) and therefore storage and data transfer needs to be planned. It is recommended to work with native binary outputs from physical circulation models, as this is often most compressed and therefore reduces the I/O overhead for IBMlib. Once the physical (and possibly biogeochemical) data set has been established, one needs to implement the physics interface in Table 2, possibly following one of the templates in the source code distribution (or recasting the data set).

For a Lagrangian study (forward, backward or connectivity simulation), an individual-based biological model, which implements the biology API in Table 1, must be selected or developed from one of the templates coming with the source code distribution. A central choice is here the necessary complexity level, at which the organism is represented in IBMlib. It is advised to start simple with the most important and robust features of the life cycle and then gradually build up the complexity level, depending on the data that is available for parameterizing the biological model. Data sources are literature, field observation data, data for related species and finally generic ecological models, e.g. size-based models [35] to fill remaining holes. Backtracking is only possible in off-line mode, because very few physical models are able to run backwards in time.

### Validation

Since the purpose of this paper is to demonstrate the IBMlib framework, we will be concerned with implementation validation aspects rather than scientific validation aspects, which belongs to papers presenting scientific results. The IBMlib core code rarely changes and has been tested by application in several projects; it is known to reproduce analytical results numerically, when available (e.g. correct particle trajectories when artificial simplified current fields are applied). The IBMlib distribution comes along with an auto-test suite to probe code integrity and basic correctness of the code building. IBMlib supports good usage practice by saving input parameters in a single input file and storing the IBMlib revision version number in output for full reproducibility. IBMlib follows a good coding standards, as stated in the user manual. When introducing new physics/biology modules, careful testing is performed to assure implementation correctness. For new biological modules, tests are very contextual and opportunistic, but as a minimum, reproduction of known limits/reference points, using synthetic hydrography, should be accomplished. The IBMlib distribution comes along with physics/biology test modules, e.g. synthetic hydrography and passive particles to provide idealized situations here expected results can be reproduced, as a basic implementation test.

### Performance

To assess the performance envelope of IBMlib, we performed an indicative benchmark on a low-end desktop for a typical Lagrangian simulation. The technical configuration is detailed in S8 Appendix. Table 3 illustrates typical Lagrangian simulation duration and computer memory needs for a simulation tracking an ensemble of particles for 224 days, which is typically the upper limit for the pelagic phase of most species. Wall time refers to the clock time of the simulation, i.e. CPU time, I/O and miscellaneous latencies altogether on the test hardware. Maximum virtual memory usage refers to the peak virtual memory allocated during the simulation job. In Table 3 we see that the I/O part roughly is 1.5 hours of the simulation and buffers needed to represent realistic physics are typically 125 MB. Particle propagation approximately totals 0.07 sec per particle, whereas the look-up and boundary enforcing overhead is roughly 0.15 sec per particle for the full simulation, while memory usage 80 Mb per million particles. If particles are more complex (with more internal states associated with internal dynamics), particle propagation cost will increase along with the memory usage per particle. The break-even point between I/O overhead and particle dynamics for off-line simulations is approximately 25000 particles for realistic physics, i.e. when the ensemble is larger than ∼ 25000 particles, performance is limited by the implementation of the particle dynamics. Usually, Lagrangian simulations need more than 25000 particles to sample the biological situation with sufficient statistical accuracy.

**Table 3:** Benchmark results. Benchmark of simulation for different number of particles released and different physical models applied. For the HIROMB Baltic Model (HBM) data set, see [20–22].

physics	particles	wall time [sec]	maximum virtual memory [Mb]
linear_flow_field	100	10.1	2.7
linear_flow_field	10000	722.5	3.4
linear_flow_field	1000000	74320.3	82.8
HBM	100	5562.2	125.2
HBM	10000	7689.3	125.9
HBM	1000000	231461.1	205.3

## Usage examples

Below we provide some representative usage examples of IBMlib to illustrate its versatility. No plotting tools are integrated or shipped along with IBMlib. The rationale is that personal flavors are very diverse and too much work is involved in maintaining a suite of plotting tools. For the same reasons IBMlib does not come along with a GUI to build IBMlib and configure/start simulations.

### Lagrangian simulations using different circulation models

**Fig 4:**
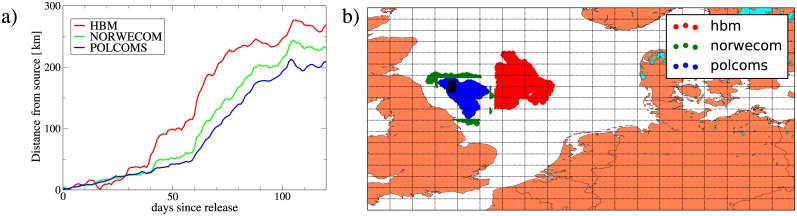
Comparison of drift of herring larvae released in autumn 2000 in the North Sea. Simulations using operational setups of different circulation models: HBM, NORWECOM and POLCOMS. (a) Average distance from spawning area after initial day-of-release of larval distribution (b) Snapshot of larval distributions 50 days after time of release. Black box indicates spawning area following [36].

Flow fields are the primary input to Lagrangian models and exploring consequences from flow field uncertainty is a primary research topic to establish the realism of Lagrangian simulations. In practice, such comparisons are often difficult to establish, because the same situation has to be represented in different Lagrangian frameworks, giving rise to hidden differences, like boundary interactions and numerical issues from data interpolation and time integration of dynamical equations and randoms numbers; this is especially true, when Lagrangian particles are not passive. IBMlib offers the possibility of a direct comparison of different flow fields for the same Lagrangian setup to draw the uncertainty envelope implied by flow field uncertainty. To illustrate this we consider the dispersal of herring larvae in the North Sea, described by Eqs (1) and (2). The spawning grounds of North Sea herring stocks are well-mapped [36, 37]—here we consider larval dispersal from the eastern patch of the Banks spawning grounds from Fig 1 in [36] as a demonstration case, simplified to a black box in our Fig 4(b), where Lagrangian particles representing larvae were released through the entire water column. Particle trajectories were integrated forward using second-order Runge-Kutta with a time step of 300 s. Duplicate simulations with this setup were performed with three physical models offline: the HIROMB Baltic Model (HBM) [20–22], the NORWegian ECOlogical Model (NORWECOM) [25, 26] and the Proudman Oceanographic Laboratory Coastal Ocean Modelling System (POLCOMS) [27, 28] implemented for the NW-European shelf. Physics data from HBM and NORWECOM were stored at hourly resolution, whereas POLCOMS are daily-averaged flow-fields. Fig 4(a) shows average distance from spawning area after day-of-release. Models fairly much agree up to 39 days after release; after this, there are significant divergence, with HBM most noticeable branching out. The figure shows that small-scale fluctuations between models are highly correlated, probably corresponding wind-driven events. Fig 4(b) shows a snapshot 50 days after release—here the HBM divergence is visible as stronger east-ward dispersion. Fig 4 illustrates that in this case there is qualitative agreement between Lagrangian simulations with different physical data sets, but for quantitative assessments one likely needs a physical model ensemble to estimate the uncertainty on results coming from differences in physical data sets. IBMlib makes such an ensemble run a routine exercise, once the interfaces to physical data sets have been established.

### Active particle motion

The null model in Lagrangian simulations is usually passive particles (*w*_*i*_ = 0 in Eq (1)), as applied in the example above, and often studies does not go beyond this level. However, the dispersion of larvae in the ocean is regulated by both active particles motion [38] and local ocean currents, and especially if time scales of motion and currents match, resonant transport phenomena [39] may occur, which are indeed used by some organisms in their life cycle [40]. Fish larvae can indeed display a wide range of active behavior motions, such as diel vertical migrations (DVM), gradient following and schooling [38] and changes in the behavior can have important consequences on connectivity between different regions. IBMlib easily allows to add arbitrary active particle motion which is overlaid onto passive drift.

**Fig 5:**
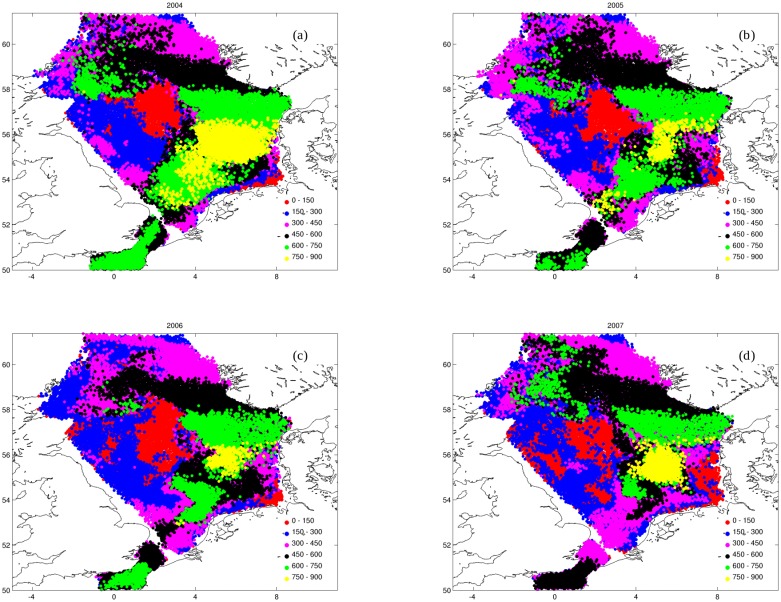
Influence of DVM active behavior. Maps of the final distribution of cod larvae exhibiting DVM active behavior for the period 2004-2007- different colors indicate the total displacement (in km) of the larvae from the spawning origin.

As a specific example we consider cod larvae in the North Sea which are transported over long distances; such transport can potentially facilitate mixing of different sub-populations hence limit genetic divergence. On the other hand different areas of the North Sea can be characterized by contrasting transport regimes (retention or dispersion) and small shifts in the spawning ground can result in large changes in transport conditions. From observed distributions of cod eggs and larvae [41, 42] we calculate a probability density function for the North Sea and English Channel that regulates spatial distribution of released particles; this release probability function represents both the observed spatial and temporal variability of cod eggs and larvae in [42], see S9 Appendix. Variability in time of particle release was simulated assuming temperature-dependent effects on the maturation of adult cod, similarly to a method used to identify spawning period for other fish species in the North Sea [43]. The transport of cod eggs and larvae were simulated from January to the end of May in different years (2004–2007). After release, cod eggs have a development period of ca. 2 weeks when they are not feeding and exposed to passive dispersion. When entering the larval stage (hatching at size of 3 mm) larvae start growing according to a simple linear function of age that closely reproduce observed data on cod larvae growth in the North Sea [44]. Two weeks after hatching cod larvae can also employ DVM and we assume that they swim close to the surface (10 m) at night and dusk while deeper (40 m) during the day. The hydrodynamical operational model used in this example is the NORWECOM model. Particles trajectories were integrated forward in time using the Runge-Kutta 2nd order algorithm with a time step of 200 sec. Although exposed to an important year-to-year variability, simulations of larval dispersion in the North Sea show consistent patterns (Fig 5). An area of low dispersion is present for all simulations in the central North Sea with larvae spawned around (56°N, 3°E), which are often displaced less than 50 km in simulated period Jan-Jun for all years 2004-2007. The more we move towards the boundary of the basin the more larvae are subject to transport, typically with displacement within 200 km from the spawning origin. The largest transport and final displacement is in correspondence of the German Bight and the Jutland currents (south and east North Sea) where larvae generally travel more than 350 km from their spawning ground. IBMlib allows to work with extended release in space and time and each larvae has a unique identity used for tracing its origin and life history.

### Habitat connectivity

We have studied connectivity for several more or less sedentary species [45–47], where connectivity between habitats is established by early pelagic life stages. For plaice, spawning habitats does not necessary correspond to settlement habitats. Connectivity (as set by the larval pelagic phase) was calculate according to Eq (3), and the biology and physics setup was as described in [47, 48]. Ontogeny and behavior were parameterised following [49] and [50], with dynamic stage durations, depending on ambient water temperature.

**Fig 6:**
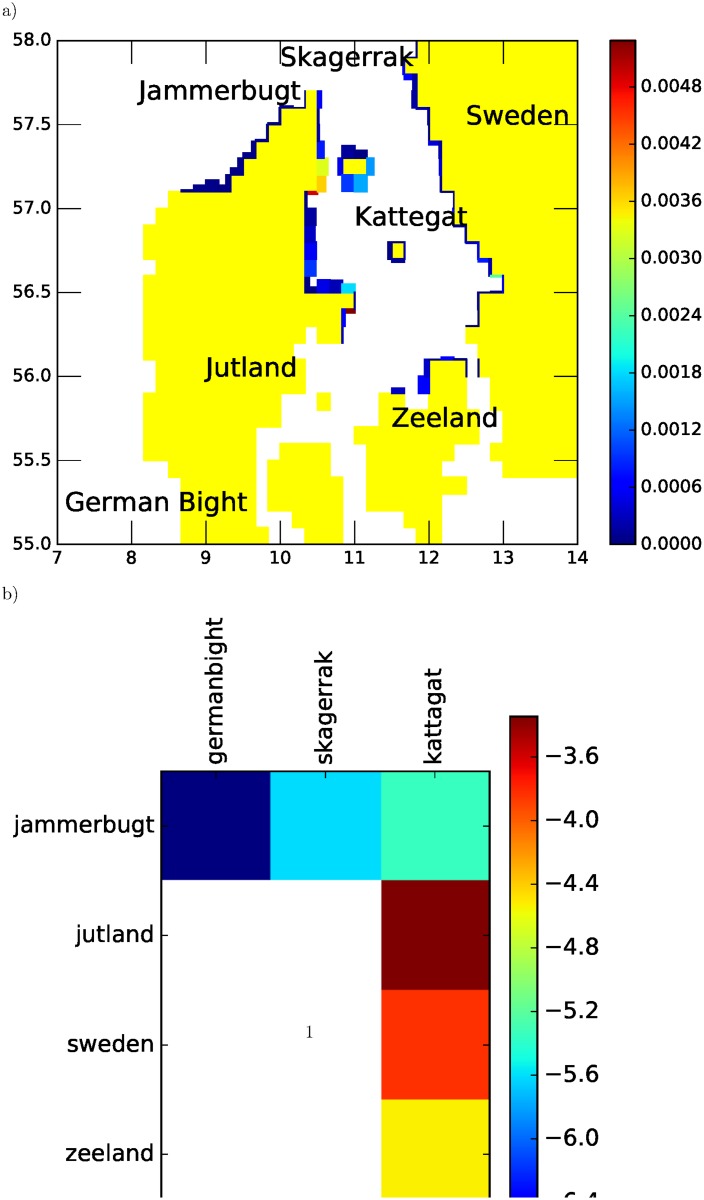
Plaice connectivity in Skagerrak/Kattegat. (a) Normalized settlement intensity for plaice in Skagerrak/Kattegat of Denmark in 2013 with map resolution (∼ 10 km) corresponding to the underlying physical model HBM. Color intensity is settlement per spawning per area (b) Connectivity matrix on log10 scale for plaice larvae spawned in German Bight, Skagerrak, Kattegat settling in Skagerrak/Kattegat. Spawning area are columns, settling area are rows.

Fig 6(a) shows normalized settlement intensity at coastal nurseries for plaice in Skagerrak/Kattegat in 2013, using the HBM data set [20–22] for physics, accessed by the IBMlib physics interface. Normalized settlement intensity means it is assumed that contributing spawning grounds (see Fig 4 in [48] have same spawning intensity per area. Habitat suitability as nursery ground is indicated by rectangle width perpendicular to the coast line, based on topography and sediment data [51] and biological knowledge [48] of habitat preferences. Fig 6(b) shows the regionalized connectivity matrix between pelagic spawning areas and coastal nurseries; we see that settlement in Kattegat/Skagerrak is mostly local, but with North Sea German Bight spawning contributing a little to settlement in Jammer Bugt in the southern part of Skagerrak, as also observed by [50]. More distant North Sea spawning sites like Dogger Bank has negligible contribution to settlement in Kattegat/Skagerrak. Interestingly, locally spawned larvae in the Jammer Bay are retained in the Skagerrak/Kattegat system in this year. The underlying assumption in this plot is that spawning intensity per unit area is the same for all spawning locations. However, if the spawning intensity is higher in some areas (which is likely the case in the North Sea), North Sea spawning may still impact population dynamics in Kattegat/Skagerrak, because the recruitment contribution is the product of transport probability and spawning intensity. We also see that settlement is quite variable along the coastal nurseries; neighboring habitats are correlated, but there are also noticable settlement hot spots. Repeating the calculation for other years than 2013 we find that the connectivity is far from static, but may vary significantly. This is a strong example of the match-mismatch dynamics [52] in a biological system. Even though these types of calculations are associated with uncertainty in the biological parameterization, they herald the variability envelope that can be expected in nature. IBMlib has a task module that automatically computes the connectivity matrix *T*_*ji*_ from a Lagrangian simulation by Eq (3).

### Ocean parameter assessment

*In situ* observations of ocean properties rarely cover the needs in marine biology, where one tries to correlate ecological processes with local ocean properties. One is left with the possibility of making dubious interpolations between sparse *in situ* observations with various uncertainties and biases; alternatively, one may access these ocean properties via high resolution output fields from properly validated ocean circulation models, giving full and consistent coverage in space and time. IBMlib is a very versatile facilitator, since it is very easy to extract ocean properties using the physics API in Table 2.

**Fig 7:**
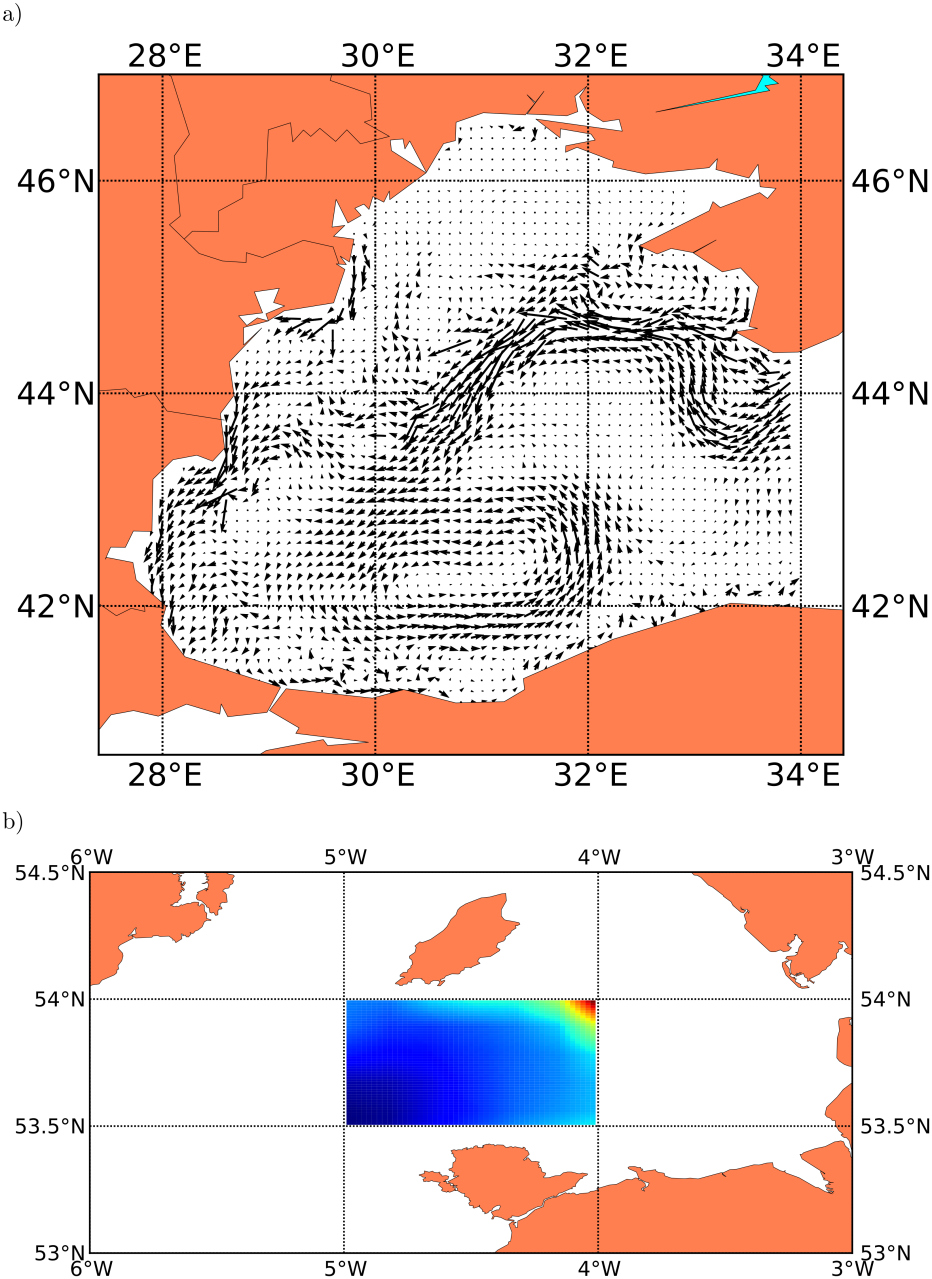
Ocean parameter assessment. a) Quiver plot of the average surface currents in the Black Sea on 01 Jan 2004, extracted from a BIMS-ECO data set by IBMlib. b) Micro zooplankton density in ICES statistical rectangle 36E5 covering 5°W to 4°W and 53.5°N to 54°N in the Irish Sea, averaged over the upper 10 meters through April 2004, extracted from a POLCOMS+ERSEM data set by IBMlib.

As an example of simple ocean parameter assessments we show in Fig 7(a) a quiver plot of the average surface currents in the western Black Sea on 01 Jan 2004, extracted from a Black Sea Integrated Modelling System (BIMS-ECO) data set [53, 54] using the current interpolation from the IBMlib physics interface to the data set. Here the well-established hydrographic mesoscale features [55] like the rim current and stationary Western Gyre are clearly visible. Fig 7(b) show the micro zooplankton density in ICES statistical rectangle 36E5 in the Irish Sea, averaged over the upper 10 meters through April 2004, extracted from a POLCOMS+ERSEM data set [27, 56] using the zooplankton interpolation from the IBMlib physics interface to the data set. These examples illustrate that extraction and post processing of ocean parameters from hydrographical data sets is easy and flexible.

**Fig 8:**
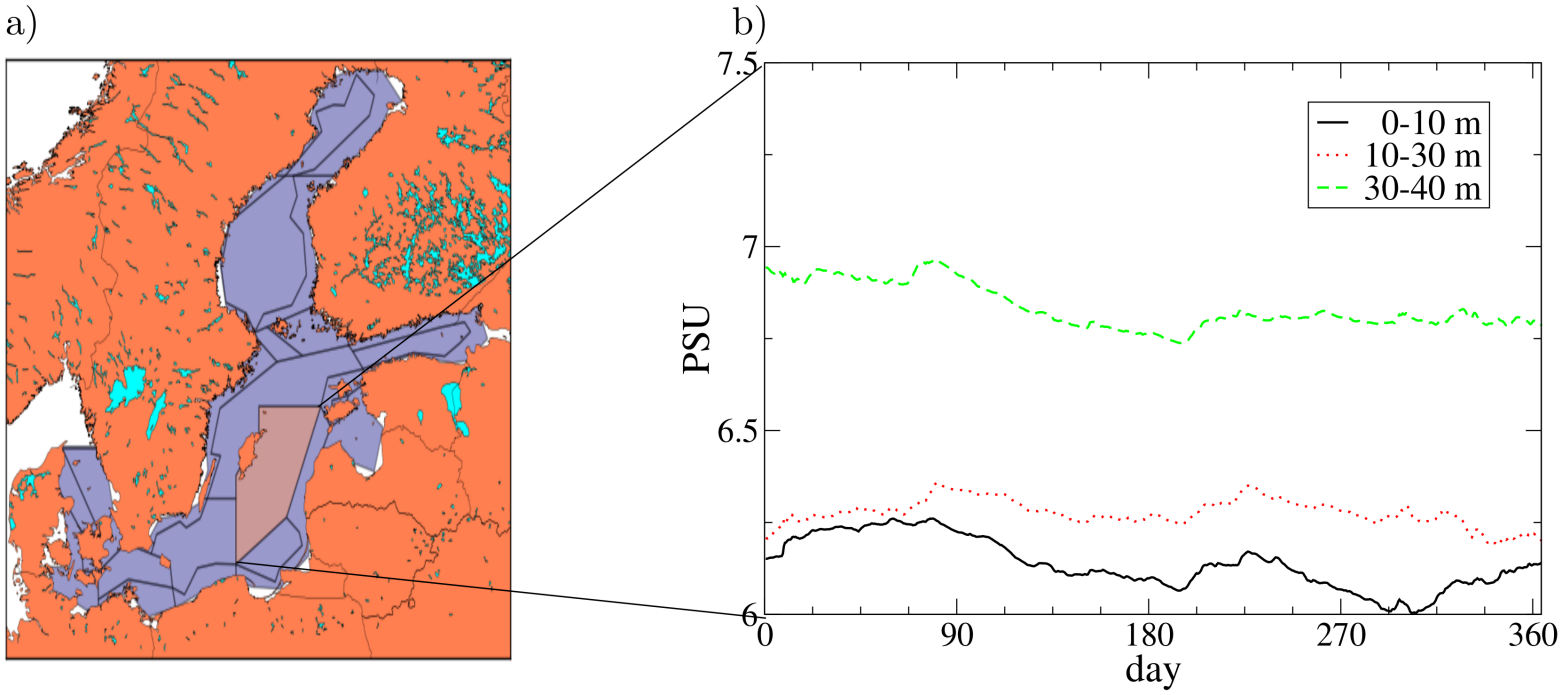
Input data generation for spatial biological modelling. a) Atlantis habitat polygons for the Baltic setup [57]. b) Time series of salinity for 3 vertical strata in 2005 for a selected habitat polygon (brown, indicated by connecting lines to two box corners).

As an example of a more complex ocean parameter assessment task, Fig 8 illustrates input data generation for the Atlantis model [32], which is a polygon-based end-2-end ecosystem model, as set up for the Baltic region. Fig 8(a) shows the habitat boxes in the Baltic setup [57] of Atlantis model, where each box needs physics/biogeochemical initial conditions as well as forcing time series from a physical model. IBMlib has a task module that reads an arbitrary Atlantis setup and produces the necessary initial and forcing data to run the Atlantis model. Fig 8(b) illustrates a time series of salinity for a selected box of the Atlantis model grid. IBMlib easily allows to compare physics uncertainty by simply changing to physics modules.

### Eulerian dynamics

**Fig 9:**
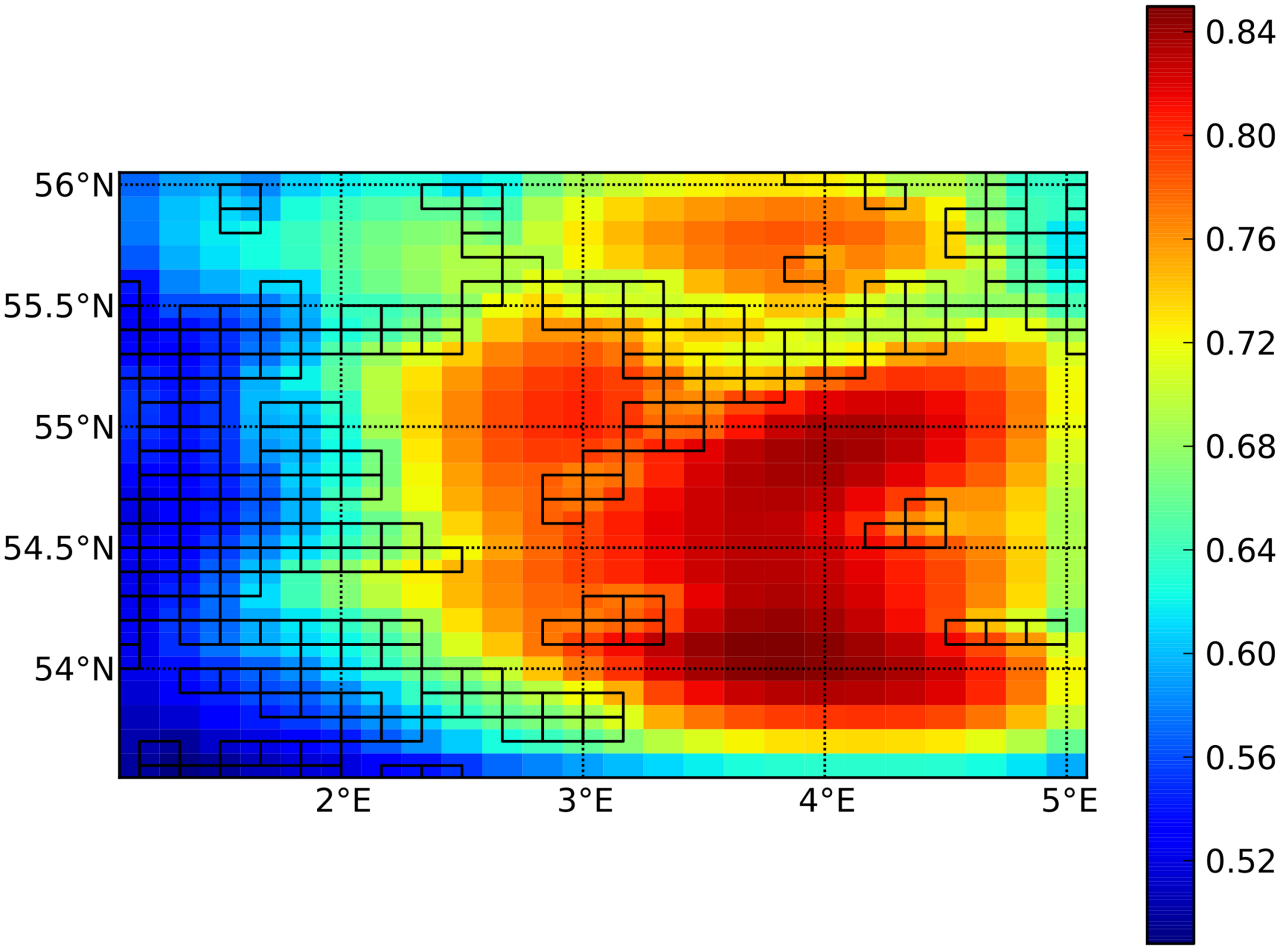
Local down grazing modelling. Local down grazing of zooplankton in the Dogger Bank area of the North Sea, simulated by IBMlib in the Eulerian configuration. Color intensity shows time-averaged zooplankton density on scale of the saturation level. Boxes are sandeel foraging habitats populated with a biomass at a level corresponding to regional stock assessment. The biological and physical setup are as in [58], except that the simulation is for 2009.

Eulerian simulations in a box geometry using IBMlib was recently demonstrated in a work studying spatial grazing depletion scales of zooplankton by sandeel distributions [58] in the Dogger bank area in the North sea with a highly simplified zooplankton model for $\hat{G}C$ in Eq (4). Fig 9 shows the time-averaged spatial heterogeneity of zooplankton caused by localized grazing when modelled with IBMlib in the Dogger bank area. Down-grazing in habitat areas and spatial recovery scales are clearly visible in this plot. The zooplankton production operator was modelled a a simple logistic growth and local grazing estimated from biomass distributions in known sandeel habitats. The biological and physical setup are as in [58], except that the simulation is for 2009. Current fields for the advection-production equation is this work was generated from the HBM model [20–22] using the IBMlib physics interface. Such applications explore the effect of dynamic model closure for biogeochemical models [59], which may be needed to improve the skill of these models in the future. The extensions needed to the basic IBMlib layout in Fig 1 are elaborated in S5 Appendix.

### IBMlib as a compliment to traditional biological investigations

**Fig 10:**
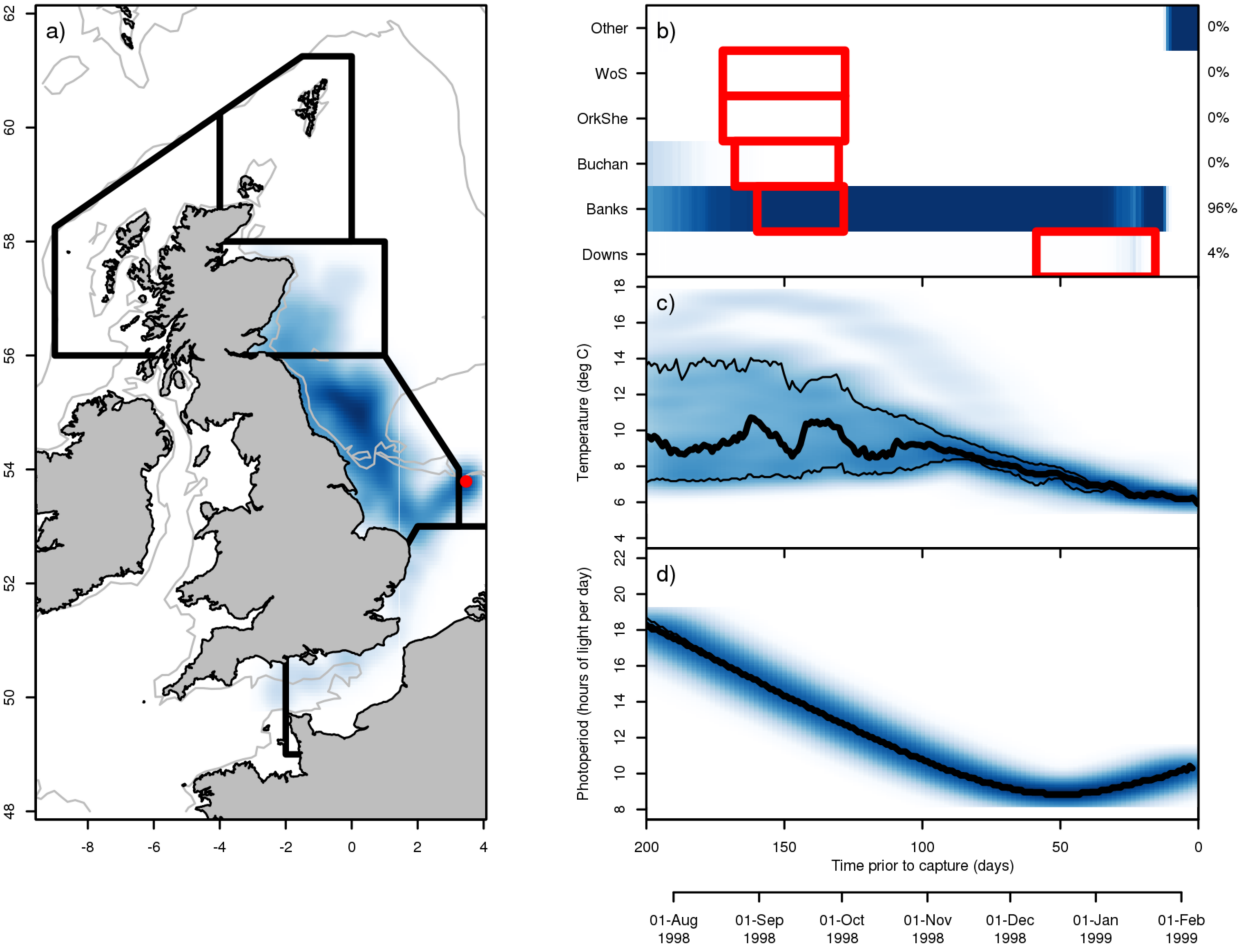
Reconstruction of early life histories. Reconstruction of environmental history for a single larva using hydrographic backtracking in IBMlib. (a) Density of back-tracked particle trajectories (blue coloring: darker shades indicate a higher density) released from the position of larval capture (red dot). The 50 and 200 m isobaths are shown for reference (light grey lines). Thick black lines denote the spatial regions associated with each herring spawning component in the North Sea. (b) Assignment of larvae to a spawning component: darker blues indicate a higher proportion of larvae. The known spawning periods of each component are indicated by the red boxes and the estimated contribution of each component to a haul is indicated at right—this larva is clearly of ‘Banks’ origin. (c) Temperature history of the larvae; median value (thick black line) and central 95% confidence region (thinner black lines). (d) Photoperiod (daylight hours per day) experienced by the larva.

The particle tracking features of IBMlib have been used as a tool to compliment more traditional biological investigations and data. Applications range in complexity from simple checking of underlying assumptions to outright integration into analyzes on an equal footing with biological observations. A common problem when working with ichthyoplankton data is relating the eggs and larvae observed in one spatial location to the position where they were actually spawned by their parents. IBMlib was used in backtracking mode by applying Eqs (1) and (2) in reversed time. to estimate the distribution and variability of distances drifted by eggs and larvae up to the point of observation. The authors found that larvae drifted less than typical ranges of spawning areas. By checking this basic but fundamental assumption, IBMlib was therefore able to facilitate further analysis. IBMlib has also been used to provide inputs to directly compliment biological observations and thereby generate unique insights. Particle back-tracking was performed for all larvae observed in these herring surveys using IBMlib to reconstruct an estimate of the individual temperature history of each larva. When coupled to lab-derived relationships between temperature and larval growth, these histories could then be used to estimate the age of the individual larvae observed. When then integrated over the entire set of observed larvae, it was possible to recover an estimate of the mortality of the larvae by year and spawning component [60]: clear differences between the components could be seen together with a strong trend in recent years. A second analysis of herring larval observations [61] took this concept even further by providing information on an equal footing to biological analyzes. In addition to thermal history, IBMlib also provided photoperiod and potential spawning grounds for observations of late herring larvae in the North Sea (Fig 10). The examples above illustrate typical usages; there are also more advanced examples, like online coupling with an ocean circulation model (see S3 Appendix) and complex energy budget models for organism growth (see S4 Appendix).

## Discussion

The usage examples provided in this paper are limited to biological examples; however the biological interface (Table 1) provides a completely generic protocol that naturally includes abiotic tracers, such as debris and pollutants (e.g. degradation would be handled by update_particle_state and buoyancy would be handled by get_active_velocity in the biology API in Table 1).

The template philosophy of IBMlib works quite well, especially when one considers the very diverse application examples above handled by a single overall framework. The template philosophy is pragmatic, so that standard tasks (passive simulation, connectivity, data extractions) are supported by a fair range of common input options so IBMlib offers a good compromise between easy-use and ultimate flexibility by tailoring source code for specialized usage. On the other hand, we can see that working directly in Fortran (or other high performance languages) often represents an irrational mental barrier to scientists and students without quantitative backgrounds. Therefore a future development line of IBMlib is also to couple to user friendly front ends, where IBMlib becomes the back end engine running the actual calculation, and the front end prepares/runs/postprocesses the calculation. This may include a markup syntax to specify a biological individual-based model. Data handling and storage can also be a challenge, particularly for users that are not used to working with the volumes of data commonly associated with oceanographic and climate model outputs. However, the modular design of IBMlib can be used to circumvent some of these problems. For example, in regions where tidal currents are important, the offline database can potentially be separated into a high-frequency regular signal and lower-frequency variation, thereby dramatically reducing the required time resolution and storage space. Because the physical provider in IBMlib hides the exact details from the rest of the framework, recombining the two components is relatively straight forward. Similarly, physical providers can also be envisaged that retrieve their data directly via the internet (e.g. via OpenDAP) rather than from a locally-stored offline database: again, as long as the provider has the correct interface to the rest of the framework, the details of its implementation are largely irrelevant. Extending the suite of physical providers to include such functionality represents a clear future development direction for IBMlib.

It is our experience that the biology API of IBMlib gives sufficient flexibility to handle all encountered needs of process representation, where biological knowledge at the individual level should be translated into Fortran code. The customizable state_attributes class allows the storage of any auxiliary information and update_particle_state is allowed to call any auxiliary functions to implement a given process representation

Our main experience is that realistic topography and boundary condition enforcement is the most difficult part to implement efficiently and consistently, if a new physics grid type is to be added. Particles can bounce complex coast lines in many exotic ways, and if boundary condition enforcement has a weak point, it will be exposed, since the implementation is typically challenged by ∼10^10^ attempts each simulation. Some Lagrangian implementations solve special cases of coast line interactions with pragmatic fixes, but this may lead to artificial distribution of particles in coastal zones. Another important issue in adapting the physics API corresponding to a new data set is precise communication with the operators of the circulation model producing the input data set for IBMlib. Possibilities of misunderstandings are numerous in relation to e.g. data format, grid layout, nomenclature, units, annotations, implicit assumptions and association to measurable hydrographic parameters, and many polite and carefully formulated follow-up emails should be anticipated. Consequently, the implementation validation for the physics API is very important, as outlined earlier. Alternatively the physics data provider can recast data into a specific format supported by an existing IBMlib physics API. Such a service would need a staff effort and is also error prone to format misunderstandings on the physics provider side; additionally, information loss is possible due to data truncation and interpolation.

A difficult aspect of model coding is hitting the right abstraction level; too high abstraction level gives voluminous, slow code that is difficult to comprehend, which eventually slows long-term model development. Too low abstraction level gives initially quick and compact code that soon, however, needs to be hacked making it difficult to understand (and validate), which again slows (or kills) model long-term model development. Based on previous experiences we believed we have chosen a good abstraction level in IBMlib, as ultimately documented by the longevity of the code. IBMlib is not a monolithic construction, but a distributed framework that contains disposable elements that can exchanged/augmented/circumvented when the unpredictable pathways of science and funding goes in directions not anticipated years ago.

## Conclusions

In the present paper we have demonstrated a versatile framework for conducting Lagrangian modelling of marine organisms in realistic, variable physical environments. The easy-coupling scheme makes our framework ideal for physical/biological model ensemble runs, which will become more and more important in the future. A core emphasis of our accumulated code development has been adherence to modern coding principles and code structural considerations aiming for a good balance between efficiency, simplicity and reusability of our code. These rationales guiding our code development has resulted in a robust code structure that has survived 5+ years and is still developing in many directions. Our main experience is that realistic topography and boundary condition enforcement is the most difficult part to handle efficient and consistently, because the implementation is typically challenged heavily in a simulation, and strict definition and implementation of the API is essential. Most commonly, Lagrangian simulations are performed with a time series of the 3D hydrography as input. For Lagrangian simulations, the break-even point between I/O overhead for loading hydrography into the computer and performing particle dynamics is typically around *N* = 25000—above this, computational load is dominated by particle dynamics scaling. Usually, Lagrangian simulations need more than 25000 particles to sample the biological situation with sufficient statistical accuracy. Therefore Lagrangian simulation implementation must be numerical efficient and therefore a high-performance coding language like Fortran or C/C++ is needed to achieve optimal numerical performance.

## Supporting information

S1 Appendix Software availability. (PDF)

S2 Appendix Connectivity. (PDF)

S3 Appendix Online mode. (PDF)

S4 Appendix Growth energy budget / multi stage models. (PDF)

S5 Appendix Posterior Eulerian dynamics. (PDF)

S6 Appendix Additional implementation details for physics modules. (PDF)

S7 Appendix Additional implementation details for biology modules. (PDF)

S8 Appendix Performance benchmark technical configuration. (PDF)

S9 Appendix Spawning maps for North Sea cod. (PDF)
