# Supplementary material for: A generic framework for individual-based modelling and physical-biological interaction
Source: PLoS One. 2018 Jan 19;13(1):e0189956. doi: 10.1371/journal.pone.0189956 (PMC5774699; doi:10.1371/journal.pone.0189956)
Supplement: S1 Appendix — (PDF) [file pone.0189956.s001.pdf]

S1 Software availability

IBMlib is a fully open source framework developed under the revision management system git on a permanent server hosted by github.com, see below. The download site contains information on how to install, run and modify the code. The git system keeps a complete log of all code changes.

Software name	IBMlib
Developers	Asbjørn Christensen, Patrizio Mariani and Mark R. Payne
Contact information	asc@aqua.dtu.dk
Hardware required	Linux/Unix platform
Software required	Fortran 90+ compiler
	gmake
	2 < Python < 3
	netCDF/HDF5 (optionally)
Download	https://github.com/IBMlib/IBMlib
Program languages	Fortran 90 (performance code)
	Python2 (build, pre and post processing)
	Gmake (build)
License	LGPL
Citation	When communicating results based on IBMlib, the present paper should be cited
