# Supplementary material for: A generic framework for individual-based modelling and physical-biological interaction
Source: PLoS One. 2018 Jan 19;13(1):e0189956. doi: 10.1371/journal.pone.0189956 (PMC5774699; doi:10.1371/journal.pone.0189956)
Supplement: S2 Appendix — (PDF) [file pone.0189956.s002.pdf]

S2 Connectivity

IBMlib has an integrated connectivity task module that allows to infer connectivity from a Lagrangian simulation. The flow is sketched Fig S1. The design principle is that a generic proxy class is encapsulates the state_attributes class defining the individual-level biology of the species of interest. The role of the proxy class is to manage net survival probability (in a super-individual interpretation) of the particle along with its generic settlement details. The proxy class and the actual state_attributes class can of course be merged ad hoc, but this construction limit code repetition, redundancy and efficiency, and means that the biological model is not mixed with settlement administration. The generic settlement class implements the biological API (Table 1) so the particle ensemble itself is not involved in the connectivity logging process. The state_attributes::update_particle_state method for instances participating in a connectivity calculation must accept an extended argument list; the standard argument list is (state, space, dt), where state, space are instances representing state (”self”) and space respectively, and dt is the time step which state should be propagated. The extended argument list is (state, space, dt, mortality_rate, die, settle), where mortality_rate is the computed mortality rate at this time step, and (die, settle) is logical variables where the state_attributes instance signals whether it died and, whether it wish to settle.

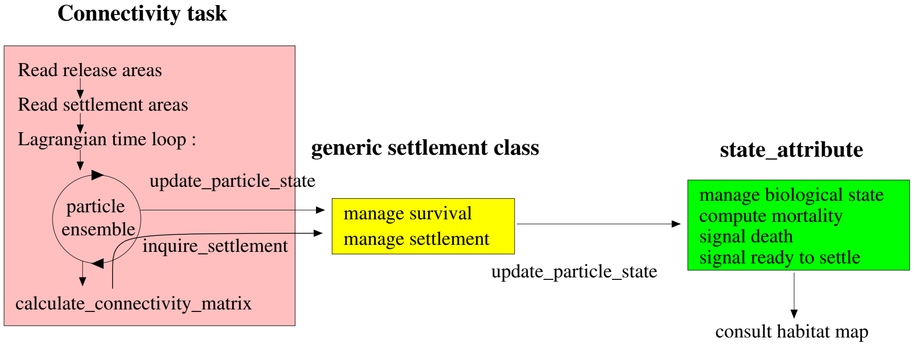

Fig S1. Flow between modules in a standardized IBMlib connectivity calculation. The state_attribute instance representing the biological processes is embedded in the generic settlement class
