# Supplementary material for: A generic framework for individual-based modelling and physical-biological interaction
Source: PLoS One. 2018 Jan 19;13(1):e0189956. doi: 10.1371/journal.pone.0189956 (PMC5774699; doi:10.1371/journal.pone.0189956)
Supplement: S3 Appendix — (PDF) [file pone.0189956.s003.pdf]

S3 Online mode

IBMlib in online mode has not yet been employed i a real study (due to lack of cases, where this has been necessary), but the API has been prepared for this and the blueprint for implementing online coupling is given below, in case a new user has study requiring this. The idea is that IBMlib should appear as plug-in, with minimal changes to host code (the physics/biogeochemistry model), so that both codes can be developed independently. The flow in IBMlib in the online configuration is sketched in Fig S2. The data exchange between physical model (host) and IBMlib (slave) may follow one of three schemes

1. Single binary file written by host (no direct data transfer). This is like an off-line coupling, just with a single file that is exchanged.

2. Static grids in host code: a single transfer call needed, where pointers to hydrographic arrays in host is passed to IBMlib. The link call link_to_host_data_(...) must be added to host once (after host initialization). This requires hydrographic arrays in host code are declared as targettable allocatables or pointers, which will be true for most cases of interest

3. Like 2) above, but this time link call needed every time before IBMlib_time_step is called

Dynamic exchange (2,3 above) requires host code is Fortran90+ (otherwise coupling may be more advanced, even though e.g. C/C++ and Fortran can often be mixed successfully).

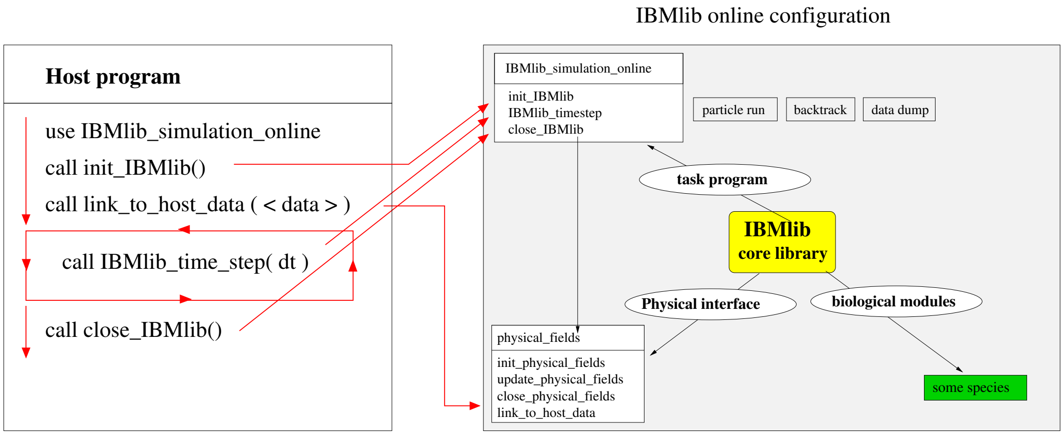

Fig S2. Sketch of IBMlib in an online configuration for a Lagrangian simulation. The left side shows the few Fortran statements that needs to be added to the host program (the physical circulation model code) to accomplish the online coupling. The sketch shows a dynamic coupling type 2 (static host grids).
