# Supplementary material for: A generic framework for individual-based modelling and physical-biological interaction
Source: PLoS One. 2018 Jan 19;13(1):e0189956. doi: 10.1371/journal.pone.0189956 (PMC5774699; doi:10.1371/journal.pone.0189956)
Supplement: S4 Appendix — (PDF) [file pone.0189956.s004.pdf]

S4 Growth energy budget / multi stage models

IBMlib comes along with templates for growth energy budget models (e.g. [62, 63]), and multiple ontogenetic stages, which often needs to be represented, when simulating early life stages. The template for growth energy budgets (Fig S3(left)) targets early life stages grazing on a continuous size-structured zooplankton spectrum and intake is modelled following optimal foraging theory [64]. It describes metabolism and growth of early life stages in relation to local food availability and ambient conditions. The template for multiple ontogenetic stages (Fig S3(right)) follows same design principle as the connectivity configuration Supporting Information S2, where a proxy class handling inter-state dynamics encapsulates biological stage classes. Biological stage classes must again accepts the extended argument list: (state, space, dt, mortality_rate, die, next), where where state, space are instances representing state ("self") and space respectively, and dt is the time step, and mortality_rate is the computed mortality rate at this time step, and (die, next) is logical variables where the `state_attributes` instance signals whether it died and, whether it wish to advance to next ontogenetic stage.

References

62. Peck MA, Daewel U. Physiologically based limits to food consumption, and individual-based modeling of foraging and growth of larval fishes. Marine Ecology Progress Series. 2007;347:171–183.

63. Daewel U, Peck MA, Schrum C. Life history strategy and impacts of environmental variability on early life stages of two marine fishes in the North Sea: An individual-based modelling approach. Canadian Journal of Fisheries and Aquatic Sciences. 2011;68(3):426–443. doi:10.1139/F10-164.

64. Charnov EL. Optimal foraging: attack strategy of a mantid. American Naturalist. 1976;110:141–151.

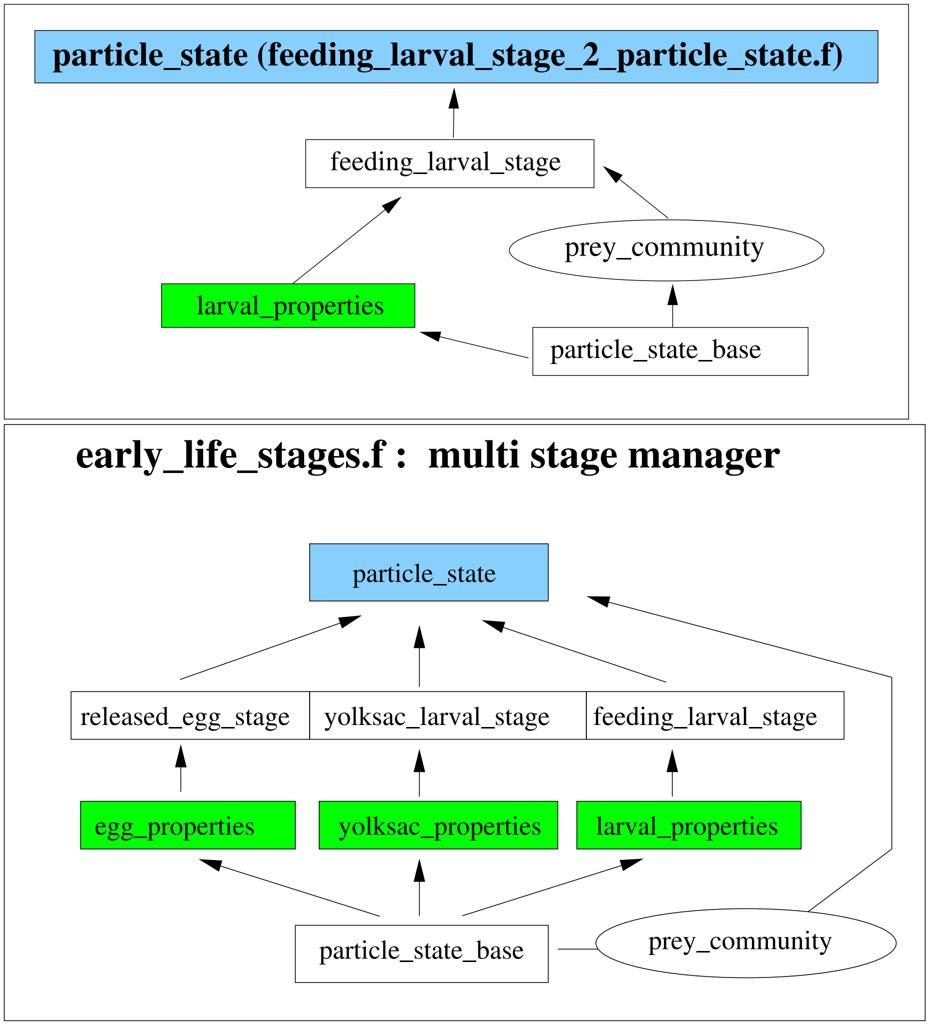

Fig S3. Bioenergetics templates. Top: optimal forager template. Bottom: early life stage decomposition
