# Supplementary material for: A generic framework for individual-based modelling and physical-biological interaction
Source: PLoS One. 2018 Jan 19;13(1):e0189956. doi: 10.1371/journal.pone.0189956 (PMC5774699; doi:10.1371/journal.pone.0189956)
Supplement: S5 Appendix — (PDF) [file pone.0189956.s005.pdf]

S5 Posterior Eulerian dynamics

IBMlib comes along with an experimental template for posterior advection-diffusion-reaction (ADR) modelling, which has been tested in a real study [58]. Posterior refers the fact that feedback on physics is not supported, whether online or offline. The IBMlib ADR module has the advantage that it can scoop out a sub region of the physical model domain and only apply the ADR module here, allowing to speed up the calculation.

Since Eulerian simulations are fundamentally different from Lagrangian simulations, the basic IBMlib configuration diagram Fig 1 is also a little different, see Fig S4. The main difference is that the biology module is replaced by a Eulerian module. The Eulerian module has a closer relation to the physics module and should be considered to be a shell around the physics module, for reasons of efficiency and because strict mass conservation must be obeyed in Eulerian dynamics, if the state variables form a closed system. The biology module becomes a point production module, which is not accessed from the task module, but only via the Eulerian module. The point-production module can contain arbitrary complex reaction processes. It should be emphasized that the physics API is still fully accessible in the task module. The Eulerian API (accessed in the task module) is given in Table 4. It shows that a class describing an Eulerian field must be provided, along with constructors/destructors and updaters. update_eulerian_field will trigger a call of update_physical_field. A folder eulerian_providers in the IBMlib distribution organizes templates for Eulerian simulations, and task_providers has an Eulerian simulation template illustrating the usage. At some point in the future a fusion between Eulerian and Lagrangian mode may be enabled, since there is no fundamental limitations on that.

Table 4. Eulerian interface in IBMlib, defining Eulerian states and dynamics.

service	type	summary
eulerian_field	derived type definition	definition of Eulerian field
init_advection_diffusion	subroutine	init this module
close_advection_diffusion	subroutine	close this module
init_eulerian_field	subroutine	constructor for eulerian_field
close_eulerian_field	subroutine	destructor for eulerian_field
interpolate_eulerian_field	subroutine	interpolate at a given position
update_eulerian_field	subroutine	Eulerian field forward step
update_eulerian_auxillaries	subroutine	updates auxillaries for Eulerian dynamics
dump_2Dframe	subroutine	dump field to file
get_data	subroutine	export pointer to data

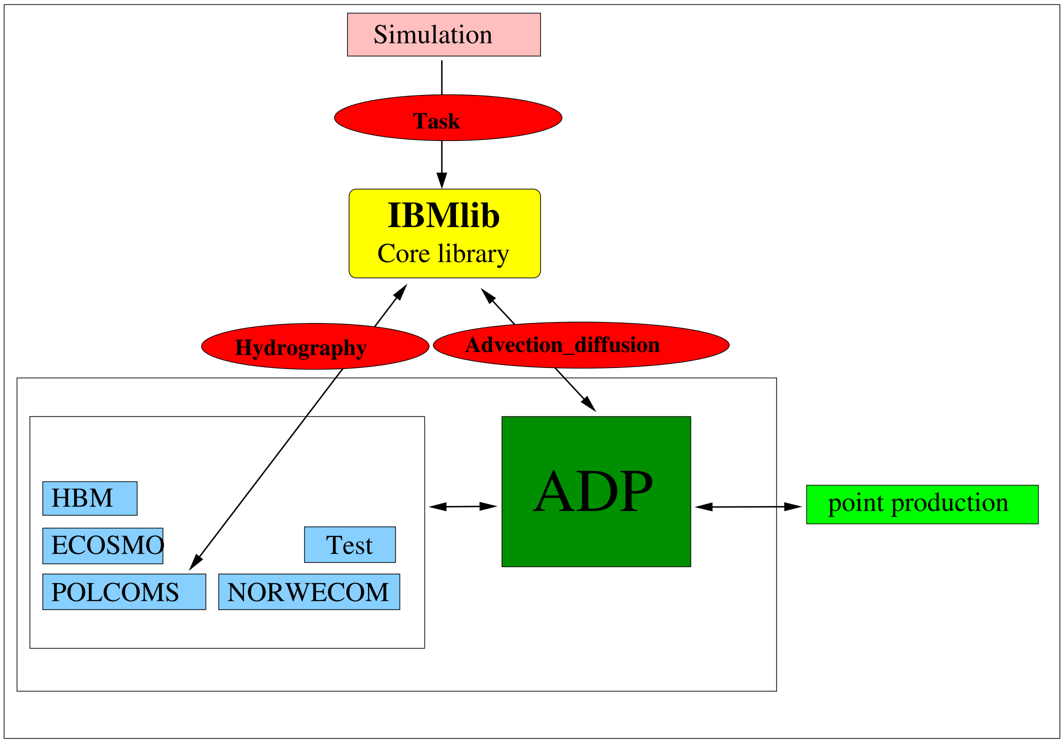

Fig S4. IBMlib in Eulerian configuration.
