# Supplementary material for: A generic framework for individual-based modelling and physical-biological interaction
Source: PLoS One. 2018 Jan 19;13(1):e0189956. doi: 10.1371/journal.pone.0189956 (PMC5774699; doi:10.1371/journal.pone.0189956)
Supplement: S6 Appendix — (PDF) [file pone.0189956.s006.pdf]

S6 Additional implementation details for physics modules

Services init_physical_fields will allocate and initialize the physics module, whereas close_physical_fields will deallocate and reset the physics module. get_master_clock and set_master_clock will access and reset the master clock held by the physics module. The physics interface works both in off-line and on-line situations; until now, no realistic simulations has been performed in on-line mode, but a template is provided for handling this situation. For on-line mode set_master_clock is disabled, since the coupled ocean circulation model will control the time progress of the coupled simulation, with IBMlib being slaved to the ocean circulation model. The explicit call update_physical_fields will synchronize physical fields in the physics module to the master clock held by the physics module which is often the most time consuming step in an off-line simulation, where the physics module loads physical fields from a data base; therefore physical fields are only updated on request to avoid unnecessary synchronizations, and physics modules also checks whether file reading is necessary at all to minimize I/O overheads, since the time step of the Lagrangian simulation and saved physical data frames is independent in off-line mode. The physics interface offers many services named as interpolate_X, which interpolates a property X at an arbitrary (wet) position, along with a status integer flagging exceptions (e.g. out-of-range interpolation attempt). interpolate_turbulence provides particle horizontal/vertical diffusivity for a particle random walk representing sub-grid scale eddies in Lagrangian simulations. interpolate_turbulence_deriv provides the diagonal of the derivative tensor which is needed for random walk step corrections to avoid artificial aggregations of particles [29] in case of spatially heterogeneous eddy diffusivity. If the underlying physical model does not provide eddy diffusivity as output, interpolate_turbulence and interpolate_turbulence_deriv should just consistently return zero, in which case particles follow pure streamlines in the currents, if they are passive, or supply a replacement model. Services interpolate_wdepth, is_wet, is_land and horizontal_range_check is a minimal set of topography-related query functions needed to perform particle dynamics. coast_line_intersection is a service function assisting enforcement of coastal boundary conditions on particle steps during particle dynamics: it detects and computes coastal impact/reflection points (if any), when moving a

particle between two arbitrary points. When implementing the physics services above it is important to adhere to a consistent geometric approximation for the topography (seabed/coastline) across services in the API. To ease implementation of the physics API to a new physics data set, an interface template (mesh_grid) exist for grids that map to a common regular longitude-latitude mesh horizontally and either z- or σ-type vertically, offering array logistics, generic 2/3D interpolators and topography query services. This template applies to most circulation models operated today. In the IBMlib root folder, a sub folder oceanography_providers provides a set of physics modules for different physical data sets coupled to IBMlib in studies until now, along with some artificial fields, e.g. constant flow fields in idealized topographies, which are very useful for testing.
