# Supplementary material for: A generic framework for individual-based modelling and physical-biological interaction
Source: PLoS One. 2018 Jan 19;13(1):e0189956. doi: 10.1371/journal.pone.0189956 (PMC5774699; doi:10.1371/journal.pone.0189956)
Supplement: S7 Appendix — (PDF) [file pone.0189956.s007.pdf]

S7 Additional implementation details for biology modules

In the IBMlib root folder, a sub folder biology_providers provides a set of biology template modules, spanning different species featuring different biological complexity, as well as basic minimal types, like generic_larvae, or testing modules each implementing the biology interface. state_attributes is a Fortran90 derived type (called a class in object-oriented lingo) with arbitrary content describing/logging all aspects of the particle beyond generic spatio-temporal properties. init_particle_state allows to allocate and initialize module data, e.g. common parameters or maps and and close_particle_state allows to deallocate biology module data init_state_attributes will initialize an instances of particle_state and possibly also set certain space attributes (like boundary conditions and mobility). get_active_velocity will return the current motion velocity vector (in relation to the water mass) for the particle, in relation to its current state and ambient conditions. update_particle_state will propagate the internal states of a state_attributes instance corresponding to a positive/negative time step. delete_state_attributes is the destructor associated with state_attributes (that should deallocate pointers, if allocated to avoid memory leakage, and reset data fields). Finally write_state_attributes print particle_state content in a readable form to stdout for debugging. Most services in the biological API has a few generic arguments; we refer to the code documentation for a listing of the subroutine call interface. update_particle_state in the biology module implements Eq. 2 by computing the internal state increment rate G_i , whereas Eq. 1, which is generic, is handled by the IBMlib core library. Running Lagrangian backtracking requires that update_particle_state in the biology module accepts negative time steps.
