# Supplementary material for: A generic framework for individual-based modelling and physical-biological interaction
Source: PLoS One. 2018 Jan 19;13(1):e0189956. doi: 10.1371/journal.pone.0189956 (PMC5774699; doi:10.1371/journal.pone.0189956)
Supplement: S8 Appendix — (PDF) [file pone.0189956.s008.pdf]

S8 Performance benchmark technical configuration

The benchmark is performed on a quad-core CPU (Intel Xeon E5430 @ 2.66GHz) with 4GB RAM, running Linux (Ubuntu 12.04, kernel 2.6.32) with IBMlib compiled by the Intel compiler (ifort Version 10.1). The benchmark compares two types of flow fields: a synthetic flow field, which is a horizontal, stratified flow field over a flat sea bed, constant temperature and salinity. The synthetic flow field does not have a look-up overhead for interpolating to particle positions and enforcing boundary conditions, nor does it utilize I/O or memory to represent physics; the other physics module is the HBM data set as described below, and the performance and resource needs are typical to realistic data sets with same spatial and temporal resolution. The offline HBM database of ocean current frames from the HBM model [20–22] used in this benchmark were stored in a database (Synology NAS DS1812+) with native spatial resolution (10 km horizontal and up to 50 wet z-layers) covering the North and Baltic Sea, with 1 hour resolution, so that one frame of basics physical data (3D currents, temperature and salinity) takes up to 12 MB storage space for each data layer. This database was loaded sequentially by the Lagrangian simulation running on the computer over a 100Mb/s shared connection. The biological part of the Lagrangian simulation was the same as detailed in Section "Lagrangian simulations using different circulation models", as well as the simulation period, however, we varied the number of particles released and the physical model applied.
