# Supplementary material for: A generic framework for individual-based modelling and physical-biological interaction
Source: PLoS One. 2018 Jan 19;13(1):e0189956. doi: 10.1371/journal.pone.0189956 (PMC5774699; doi:10.1371/journal.pone.0189956)
Supplement: S9 Appendix — (PDF) [file pone.0189956.s009.pdf]

S9 Spawning maps for North Sea cod

The emissions of the numerical particles in the Active particle motion case study of this paper are according to spawning habitat probabilities reconstructed from egg surveys [41, 42]. Fig S5 show the reconstructed spatial release probability function, which are used to modulate the number of numerical particles released in each site in the North Sea and English Channel.

Spawning time is calculated using the average integral temperature (degree days) in the upper 40m, starting from the autumn equinox and assuming a temperature threshold in the North Sea of 1280 °C·days [65]. Eggs development is temperature dependent [66]. Eggs are passively transported, while larvae are active swimmers in the vertical employing diel vertical migration between 10m during day and dusk, and 40m at night. The maximum swimming speed is proportional to body length (1 BL/s) and growth is age dependent [44].

References

65. Neuheimer AB, MacKenzie BR. Explaining life history variation in a changing climate across a species' range. Ecology. 2014;95(12):3364–3375.

66. Geffen AJ, Fox CJ, Nash RDM. Temperature-dependent development rates of cod Gadus morhua eggs. Journal of Fish Biology. 2006;69(4):1060–1080.

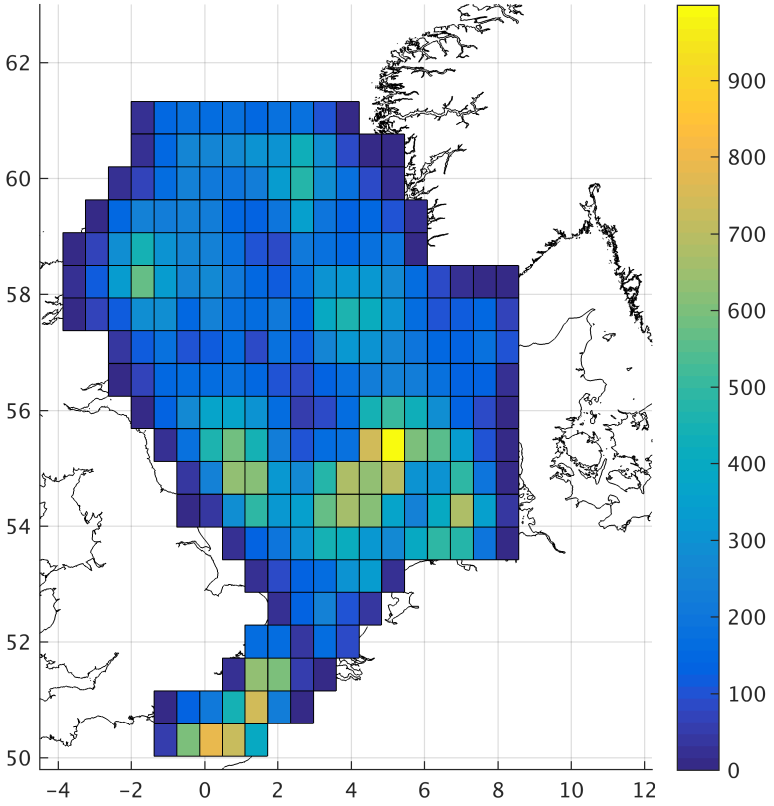

Fig S5. Cod egg spatio-temporal release probability. As reconstructeed from [41, 42]. Color scale indicates relative intensity of spawning.
